# Cohabitating in the City: A Case of Hemolytic Anemia in a Patient Coinfected With Babesiosis, Lyme Disease, and Mononucleosis

**DOI:** 10.7759/cureus.83043

**Published:** 2025-04-26

**Authors:** Maria C Tole, Maria V Perez, Yemesrach F Mekonen, Marco Bermudez, Hernando Salazar

**Affiliations:** 1 Internal Medicine, St. Barnabas Hospital (SBH) Health System, Bronx, USA

**Keywords:** anemia, babesia micoti, epstein-barr virus infection, lyme disease and other tick borne pathogens, tick, tick-borne infections

## Abstract

In the past decades, the number of tick-borne illnesses reported in the United States has increased exponentially, leading to significant human morbidity and mortality. Among tick-borne illnesses, the most frequent in the United States is Lyme, a disease caused by the spirochetal bacteria *Borrelia burgdorferi*. Coinfection with Lyme disease and *Babesia spp. *is common among ticks, rodent reservoirs, and human hosts. At an early stage, both Lyme disease and babesiosis present with flu-like symptoms, such as fever, myalgias, fatigue, headache, and malaise. Due to the nonspecific presentation of these diseases and Lyme's cross-reactivity with other viral and bacterial illnesses, there is a broad spectrum of infectious, rheumatologic, and autoimmune differential diagnoses, including Epstein-Barr virus (EBV) infection. Herein, we present the case of a 60-year-old male New Yorker who came to the hospital for nine days of watery diarrhea and recurrent fever. No rash, enthesitis, or joint abnormalities were noted on arrival. Initial lab results and imaging were remarkable for non-autoimmune hemolytic anemia, monocytosis, atypical lymphocytosis, and splenomegaly. Stool cultures and ova-parasites were negative. Treatment with doxycycline and valganciclovir was started, as IgM antibodies were positive for Lyme disease and cytomegalovirus (CMV). Due to worsening symptoms and clinical deterioration, the treatment was broadened with azithromycin and atovaquone, after which the patient experienced significant clinical and paraclinical improvement. Further studies revealed active babesiosis and EBV infection, besides a negative CMV viral load, indicating a false-positive initial result. After finishing treatment for babesiosis and Lyme disease, the patient recovered completely and remains asymptomatic to this day. Diagnosing active babesiosis, Lyme, and EBV coinfection was challenging because of the absence of erythema migrans, lack of apparent tick exposure history, and concerns for cross-reactivity. To the best of our knowledge, this is one of the first cases of coinfection among these three pathogens reported in medical literature.

## Introduction

In the past decades, the number of tick-borne illnesses reported in the United States has increased exponentially, leading to significant human morbidity and mortality. In addition to improved diagnostic technologies, the rise in tick-borne diseases corresponds to a substantial geographic expansion of tick populations throughout the country [1,2]. Among tick-borne illnesses, the most frequent in the United States is Lyme, a disease caused by the spirochetal bacteria *Borrelia burgdorferi*. Coinfections with Lyme disease and either *Anaplasma phagocytophilum* or *Babesia spp.* are common among ticks, rodent reservoirs, and human hosts [1-2].

Zoonotic Lyme disease and babesiosis have a relatively short incubation period of about seven to 14 days and one to 6 weeks, respectively [2-4]. At an early stage, both diseases manifest with flu-like symptoms, such as fever, myalgias, fatigue, headache, and malaise [2-5]. Due to the nonspecific presentation of early-onset Lyme disease and babesiosis, there is a broad spectrum of infectious, rheumatologic, and autoimmune differential diagnoses [2,4-6]. Moreover, patients with Lyme disease can exhibit antibody cross-reactivity with both Epstein-Barr virus (EBV), *Borrelia spp.*, treponema pallidum, cytomegalovirus (CMV), and rheumatoid factor, which might act as a confounding factor [7-9]. 

Mononucleosis is a common infection caused by EBV, which often presents with myalgias, fatigue, fever, pharyngitis, and lymphadenopathy. This infection represents one of the differential diagnoses for Lyme disease and babesiosis, as they share a similar clinical presentation, physical exam findings, and paraclinical alterations. For example, hepatosplenomegaly is common among patients with mononucleosis, overlapping with the clinical presentation of babesiosis [6,10]. Furthermore, about 3% of patients with Lyme disease and EBV infection develop anemia, which in the latter case can be caused by medullary aplasia or autoimmune "cold" or "warm" hemolysis [11]. Meanwhile, babesiosis is characterized by non-autoimmune hemolytic anemia secondary to red blood cell invasion by intraerythrocytic protozoans, and in rare instances, auto-immune hemolytic anemia in asplenic individuals [12]. Several cases of babesiosis and Lyme disease masquerading as mononucleosis have been reported [13-15]. However, the medical literature has documented very few instances of actual coinfection among these pathogens [15-17]. 

We present the case of a patient from New York who was found to have hemolytic anemia in the setting of active babesiosis, Lyme disease, and EBV coinfection. The diagnosis was challenging due to the absence of typical Lyme disease cutaneous manifestations, lack of apparent tick exposure history, and concerns for cross-reactivity.

This article was previously presented as a meeting abstract at the CHEST (American College of Chest Physicians) Annual Scientific Meeting on October 9, 2024.

## Case presentation

A 60-year-old man with no prior medical history presented to the emergency room (ER) with overall body weakness, profuse sweating, fever, and chills for nine days, associated with loss of appetite and watery diarrhea. A review of systems was negative for myalgias, rash, or joint pain. He had no history of recent travel or contact with domestic or wild animals. The patient denied taking any medications at home besides Tylenol. He worked as a mechanic in Queens. No other relevant medical history was documented.

On hospital arrival, the patient was tachycardic (123 beats/minute) and febrile (39.3°C). Physical examination was unremarkable; no palpable lymphadenopathy, joint effusions, enthesitis, or skin lesions were found. Mental status was preserved. He was admitted under the impression of infectious diarrhea and started on empirical treatment with ceftriaxone, ciprofloxacin, and metronidazole.

Initial laboratory tests were notable for microcytic and hypochromic anemia, mildly elevated transaminases, indirect hyperbilirubinemia, high reticulocyte count, and markedly elevated lactate dehydrogenase (LDH), suggesting ongoing hemolysis (Table 1). A series of peripheral blood smears showed microcytic red blood cells, anisocytosis, few large platelets, scarce smudge cells, and atypical lymphocytes. Parasites were not detected on three consecutive days. Significant monocytosis (28.9% of white blood cells) was noted. The Coombs test was negative, ruling out auto-immune hemolytic anemia. Spherocytosis and sickle-cell anemia were excluded, as all peripheral smears showed erythrocytes with normal morphology. Notably, the absence of considerable thrombocytopenia, schistocytes, and normal mental status rendered thrombotic thrombocytopenic purpura unlikely. Microangiopathic anemia was eliminated from consideration, as schistocytes were absent, and the patient denied any exercise, trauma, or artificial bioprosthetic devices that could cause erythrocyte damage. Other common causes of hemolysis were ruled out. Serologic tests for viral hepatitis, stool cultures, ova/parasites, and *Clostridioides difficile* (polymerase chain reaction {PCR} and toxin assay) were negative for enteropathogens. CMV IgM was positive.

**Table 1:** Laboratory studies WBC: white blood cell count, MCV: mean corpuscular volume, RDW: red blood cell distribution width (RDW), TIBC: total iron binding capacity, PT: prothrombin time, PTTa: activated partial thromboplastin time, INR: international normalized ratio, ESR: erythrocyte sedimentation rate, HIV: human immunodeficiency virus, CMV: cytomegalovirus, EBV: Epstein-Barr virus

Parameter	On admission	Follow-up (at the hospital)	Normal range
WBC (x10^3^/mm^3^)	9.0	3.4	(4.2-9.1)
Neutrophils	36.1%	25.9%	(34-67.9%)
Eosinophils	0.2%	2.1%	(0.8-7%)
Monocytes	28.9%	12.7%	(5.3-12.2%)
Lymphocytes	34.4%	58.4%	(21.8-53.1%)
Atypical lymphocytes	19%	-	0%
Basophils	0.1%	0.3%	(0.2-1.2%)
Platelets (x10^3^/mm^3^)	147	328	(163-337)
Hemoglobin (g/dl)	10.9	11.1	(13.7-17.5)
MCV (fl)	74.9	84.3	(79.0-92.2)
RDW (%)	15	19	(11.6-14.4)
Iron (ug/dl)	82	-	65-175 ug/dl
Iron saturation (ng/ml)	23.1%	-	(13-150)
TIBC (μg/dl)	255.6	-	(255-450)
Transferrin (mg/dl)	251	-	(15-50)
PT	10.8	-	9.1-11.7 seconds
PTTa (sec)	31.1	-	(30-40)
INR	1.1	-	(<1.1)
Total bilirubin (mg/dl)	9	1.5	(0.1 -1.2)
Direct bilirubin (mg/dl)	0.1	-	<0. 3 mg/dl
Serum albumin (g/dl)	2.3	3.2	(3.5-5.2)
Total protein (g/dl)	6.0	6.7	(6-8.3)
Alkaline phosphatase (U/L)	88	76	(35-104)
Alanine transferase (U/L)	78	45	(<33)
Aspartate transferase (U/L)	65	35	(<32)
Lactate dehydrogenase (U/L)	721	270	(300-600)
Creatinine (mg/dl)	1.2	0.9	(0.5-1.1)
Toxoplasmosis	<3	-	(Non-reactive)
HIV	Negative	-	(Non-reactive)
CMV IgM	74.5 (high)	-	(Non-reactive)
CMV viral load	Negative	-	(Non-reactive)
Hepatitis B virus	Negative	-	(Non-reactive)
Hepatitis C virus	Negative	-	(Non-reactive)
EBV IgM	>160.0 (high)	-	(Non-reactive)
EBV PCR	Positive	-	(Non-reactive)

**Figure 1:**
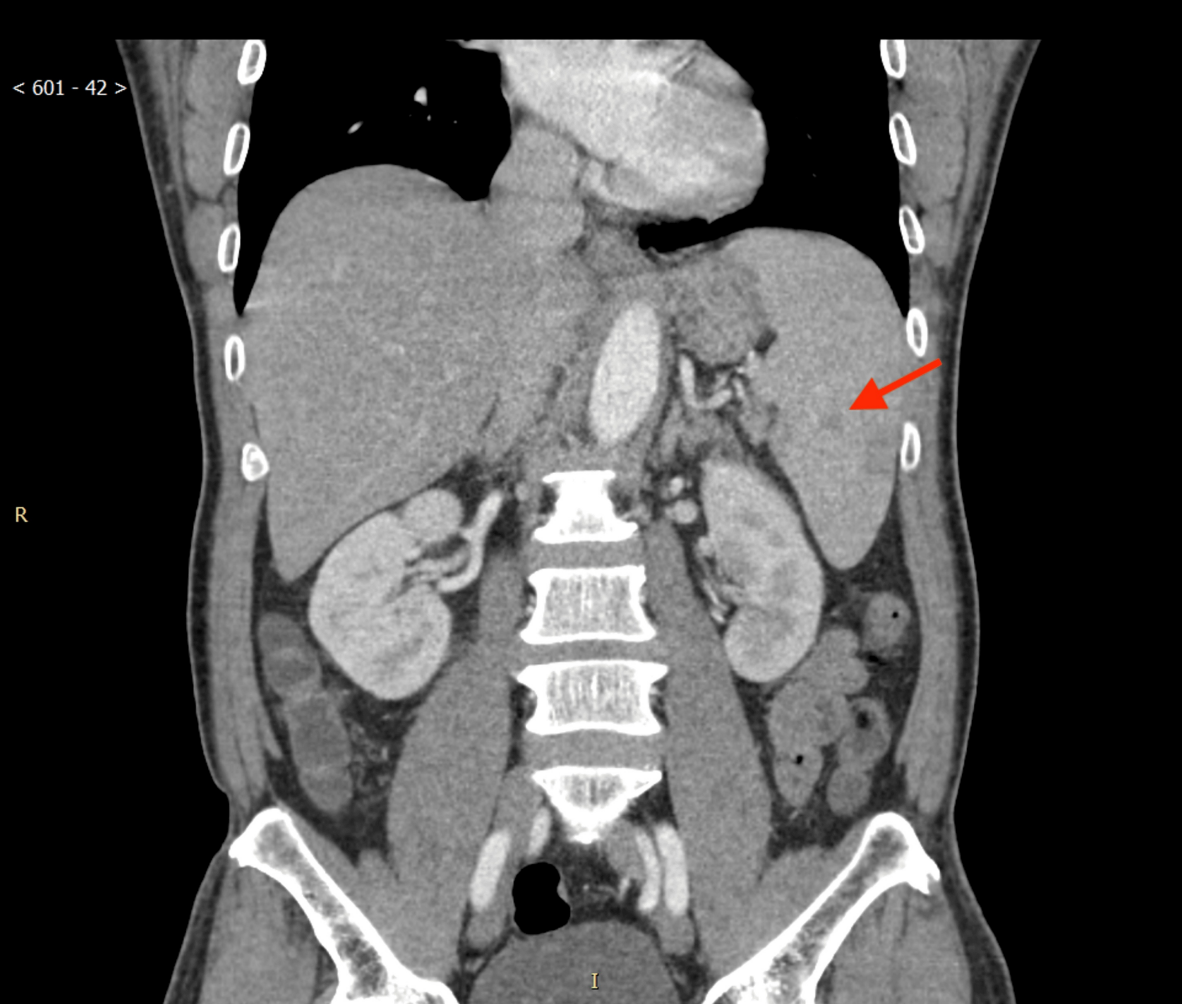
CT of the abdomen and pelvis Moderate amount of liquid stool in the colon, correlate clinically with gastroenteritis; splenomegaly (red arrow)

A computed tomography (CT) scan of the abdomen and pelvis revealed mild splenomegaly, with no strong evidence of colitis (Figure 1). No microorganisms were isolated in urine or blood cultures after five days of incubation. 

As the patient had splenomegaly and hemolysis in the setting of persistent fever, he was started on doxycycline for possible tick-borne illnesses. At the same time, ceftriaxone, metronidazole, and ciprofloxacin were discontinued. Due to persistent fever and subsequent clinical deterioration, the decision to commence empiric treatment for other tick-borne illnesses and CMV was made. The patient was started on azithromycin (500 mg every 24 hours), atovaquone (750 mg every 12 hours), and valganciclovir (900 mg every 12 hours).

Further testing revealed positive IgM antibodies for EBV and *Borrelia burgdorferi*. A Lyme disease immunoblot detected the presence of two bacterial protein bands (P23 and P41), which was insufficient to confirm the diagnosis (five bands or more needed). *Babesia* and EBV PCR came back positive, confirming acute infection. On the other hand, *Ehrlichia* PCR and CMV viral load were found to be negative. Thus, valganciclovir was discontinued and the patient continued empiric treatment for Lyme disease and babesiosis with doxycycline, atovaquone, and azithromycin. EBV was treated only with supportive measures, as the patient is immunocompetent. Two days after starting treatment with atovaquone and azithromycin the fever subsided completely and the constitutional symptoms improved considerably, after which the patient was discharged home to complete antibiotic treatment.

The patient returned for a follow-up appointment one month after hospital discharge. At that time, new blood work showed normal platelet count, improvement of anemia, kidney function, and decreasing transaminases. Lyme disease infection was confirmed due to a serologic transformation from IgM to IgG antibodies. Subsequently, the patient recovered completely and remains asymptomatic to this day.

## Discussion

In the United States, tick-borne illnesses are emerging diseases with a rising incidence that has tripled over the past two decades [17-18]. Available studies have demonstrated that in endemic areas, such as New York State and Massachusetts, up to a fifth of patients with Lyme disease are coinfected with babesiosis. However, the latter is not always symptomatic [1]. Similarly, 22% of babesiosis patients had IgM antibodies against *Borrelia burgdorferi*, independently of clinically apparent Lyme disease [1].

We present a case of triple co-infection with Lyme disease, babesiosis, and EBV in an endemic area for tick-borne infections. On hospital admission, our patient complained of non-specific symptoms such as prolonged fever, malaise, and diarrhea, which could have corresponded to a wide range of infectious diseases and a myriad of other pathologies. Initially, the chief differential diagnosis was colitis rather than a tick-borne illness, as the patient denied tick exposure and didn’t have any risk factors for zoonotic infections, including animal contact, traveling outside of the city, or engaging in outdoor activities. Also, he lacked the typical signs and symptoms of Lyme disease, such as erythema migrans and arthralgias [3]. Our data aligns with a study in Nantucket (n=192 patients with suspected tick-borne illnesses), which reported that 21% of individuals coinfected with Lyme disease and babesiosis present with flu-like symptoms without cutaneous manifestations [5].

Cross-reactivity between EBV, Lyme disease, and CMV is well known and has been amply documented in medical literature [8,15]. A study of the cross-reactivity between viral pathogens and tick-borne diseases reported that, in patients with acute EBV infection, the enzyme-immunoassay (EIA) test for Lyme disease IgM was falsely positive in 53% of cases, while the two-tier test (EIA coupled to western blot) was falsely elevated in 35% of patients. Similarly, 21-37% of patients with CMV had inaccurately high IgM titers for Lyme disease [7]. Ultimately, in patients with EBV or CMV, Lyme disease diagnosis can be confirmed by seroconversion from IgM to IgG antibodies. However, seroconversion can take weeks, and might never occur in some patients who receive early antibiotic treatment [14]. Both cultures and PCR tests are not routinely used for diagnosing spirochetal disease, due to low bacterial load and difficulty growing cultures of this specific pathogen [19]. Hence, two-tier testing remains the standard for Lyme disease diagnosis, in the setting of high clinical suspicion. In our case report, concerns for cross-reactivity and falsely elevated CMV antibodies were confounding factors that made the diagnosis challenging. At length, we confirmed Lyme disease by seroconversion to IgG antibodies after hospital discharge, as the initial two-tier testing was equivocal.

In our patient's case, the first clue of parasitic coinfection was a lack of response to Lyme disease treatment with doxycycline, which isn’t effective against *Babesia* spp., and unexpected illness severity. Laboratory results and imaging revealed splenomegaly, anemia, atypical lymphocytosis, and significant monocytosis. Although the first two can be caused by *Babesia spp.* and EBV infection, atypical lymphocytes and monocytosis in peripheral blood are typical findings of acute mononucleosis rather than a parasitic infection [5-9,10]. Furthermore, the patient presented with non-autoimmune hemolytic anemia, typically seen in Babesiosis due to red blood cell invasion by intraerythrocytic protozoa. Although Lyme disease and EBV can also present with anemia, the underlying mechanism is usually medullary aplasia or autoimmune hemolysis [12]. As babesiosis and mononucleosis have a similar clinical presentation and might exhibit cross-reactivity, blood parasitemia and/or molecular tests such as PCR for deoxyribonuclease acid (DNA) detection can help confirm actual coinfection, as was the case for our patient [10].

Finally, It has been proposed that a recent EBV infection might predispose to early disseminated Lyme disease, by altering the host immune response, especially if the patient was treated with a course of corticosteroids [14]. In our case report, the concomitant EBV infection could potentially explain why the patient (who had no other risk factors, such as old age, HIV, malignancy, or splenectomy) had an unexpectedly severe parasitic infection, associated with complications such as acute kidney injury.

## Conclusions

In endemic areas, tick-borne illnesses should always be considered part of the differential diagnoses for febrile patients, regardless of unlikely exposure to ticks in the urban setting. Among patients with suspected tick-borne disease, non-autoimmune hemolytic anemia is a hallmark of babesiosis and should warrant the prompt initiation of empiric treatment for this condition. Given the known cross-reactivity between viral, parasitic, and bacterial pathogens, molecular tests such as PCR for DNA detection are useful for establishing a definitive diagnosis of active coinfection.
